# Pharmacokinetic Analysis of Four Bioactive Iridoid and Secoiridoid Glycoside Components of *Radix Gentianae Macrophyllae* and Their Synergistic Excretion by HPLC-DAD Combined with Second-Order Calibration

**DOI:** 10.1007/s13659-017-0145-7

**Published:** 2017-11-25

**Authors:** Tian-Ming Yang, Yang-Xi Liu, Hai-Yan Fu, Wei Lan, Han-Bo Su, He-Bin Tang, Qiao-Bo Yin, He-Dong Li, Li-Ping Wang, Hai-Long Wu

**Affiliations:** 1 The Modernization Engineering Technology Research Center of Ethnic Minority Medicine of Hubei Province, School of Pharmaceutical Sciences, South-Central University for Nationalities, Wuhan, 430074 China; 2 State Key Laboratory of Chemo/Biosensing and Chemometrics, College of Chemistry and Chemical Engineering, Hunan University, Changsha, 410082 China

**Keywords:** *Radix Gentianae Macrophyllae*, HPLC-DAD, Second-order calibration, Pharmacokinetic analysis

## Abstract

**Abstract:**

An HPLC-DAD method combined with second-order calibration based on the alternating trilinear decomposition (ATLD) algorithm with the aid of region selection was developed to simultaneously and quantitatively characterize the synergistic relationships and cumulative excretion of the four bioactive ingredients of *Radix Gentianae Macrophyllae* in vivo. Although the analytes spectra substantially overlapped with that of the biological matrix, the overlapping profiles between analytes and co-eluting interferences can be successfully separated and accurately quantified by the ATLD method on the basis of the strength of region selection. The proposed approach not only determined the content change but also revealed the synergistic relationships and the cumulative excretion in vivo of the four ingredients in urine and feces samples collected at different excretion time intervals. In addition, several statistical parameters were employed to evaluate the accuracy and precision of the method. Quantitative results were confirmed by HPLC-mass spectrometry. Satisfactory results indicated that the proposed approach can be utilized to investigate the pharmacokinetics of *Radix Gentianae Macrophyllae* excretion in vivo.

**Graphical Abstract:**

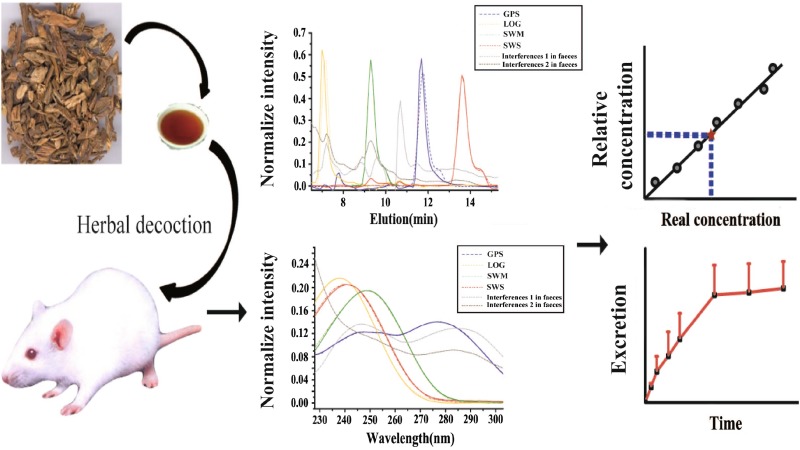

## Introduction

*Radix Gentianae Macrophyllae* belongs to the *Gentiana* genus of Gentianaceae and is widely used as a remedy in traditional Chinese medicine (TCM) for more than 2000 years [1]. The dominant bioactive constituents in *Radix Gentianae Macrophyllae* are iridoid and secoiridoid glycosides, including gentiopicroside (GPS), loganic acid (LOG), swertiamarin (SWM), and sweroside (SWS), which exhibit analgesic, anti-inflammatory, antipyretic, antirheumatic, diuretic, febrifuge, and hypoglycemic pharmacological effects for treating hypotension, rheumatism, pains, fever, and allergic inflammations [2–6].

Unlike those of chemical drugs, the therapeutic effects of TCM are based on the synergistic effect of their bioactive compounds [7]. The determination of several components can not sufficiently represent the effects of TCM [8], whereas multi-component analysis helps reveal the effect of coordination among the TCM components [9, 10]. Thus, simultaneous quantification of bioactive ingredients in a complex physiological matrix is crucial.

Pharmacokinetics describes how the body affects a specific drug after administration through absorption and distribution, the chemical changes of the substance in the body, and the excretion effects and routes of metabolites in the drug [11]. Pharmacokinetic study on multiple components is a difficult field in TCM research because of the complicated and microscale nature of the chemical components of TCM.

Based on the available literature, several analytical methods, such as HPLC-UV [12, 13], UFLC-MS/MS [14], and LC/MS/MS [15], are available for quantifying bioactive ingredients derived from *Radix Gentianae Macrophyllae* in a biological matrix. Chromatographic analysis is an effective strategy to directly determine drugs. However, baseline drift and overlapping of peaks between matrix constituents and compounds of interest often occur in the chromatographic analysis of complex samples. For eliminating the influence of interfering compounds on analytes of interest, complex gradient elution is employed to isolate analytes. Several trivial sample pretreatments and instrumental parameters require optimization, entailing considerable energy, time, and cost.

Second-order calibration is widely used in numerous scientific areas, such as food quality and safety [16–24], cosmetic research [25, 26], environmental monitoring [27–30], biochemical assay [31–37], and routine analysis [38–40]. The strategy is a good solution to the previously mentioned problems because the concentrations of individual components can be accurately obtained even in the presence of uncalibrated interferences, that is, “second-order advantage.” Pharmacokinetic research on TCM is always performed using chromatographic technique, which offers advantages of powerful separation and analysis capabilities. However, an extremely complex gradient for sample separation and specific preprocessing procedures to optimize an internal standard is difficult to develop [41–44]. To the best of our knowledge, HPLC with a diode array detector (DAD) coupled with second-order calibration based on alternating trilinear decomposition (ATLD) has yet to be reported in drug excretion studies. Simultaneous determination of TCM bioactive substances in urine and feces samples after oral administration is challenging because of complex chemical components and trace amounts of bioactive ingredients. Therefore, researchers always use complex sample pretreatment, which require time and material and financial resources. By contrast, this practical problem can be potentially resolved through the development of chemometrics. The detection of the four bioactive components (GPS, LOG, SWM, and SWS) in *Radix Gentianae Macrophyllae* in urine and feces samples is influenced by unknown interferences. Furthermore, with the aid of second-order calibration, which maximizes the collected information in multi-way data arrays, the separation capability of routine chromatographic-based techniques can be enhanced by employing “mathematical separation” to partially substitute for “physical and chemical separation” [45, 46].

In the present work, a new analytical strategy was developed by employing HPLC-DAD coupled with second-order calibration based on the ATLD algorithm to simultaneously and quantitatively characterize the synergistic relationships and cumulative excretion of the four bioactive ingredients of *Radix Gentianae Macrophyllae* in vivo. Root-mean-square error of prediction (RMSEP), *t* test, figures of merit (FOMs), including sensitivity (SEN), selectivity (SEL), limit of detection (LOD), and limit of quantification (LOQ), and reproducibility of inter-day analysis, were used to statistically validate the approach. In addition, HPLC-MS was used to evaluate the performance of the proposed approach. All results were satisfactory, indicating that the proposed strategy was simple, accurate, reliable, and time saving. The proposed strategy offers several advantages compared with other published methods: first, the combination of chemometric method with HPLC-DAD is originally applied to comprehensively quantify the four bioactive ingredients in most biological matrix systems. Second, the introduction of second-order calibration enables the separation of analytes in complex matrices in the same isocratic mode and without tedious pretreatment, thus simplifying the analysis procedure. Finally, widely used TCM, such as *Radix Gentianae Macrophyllae*, can be simultaneously analyzed under the same isocratic chromatographic condition. This approach provides a new foundation in clinical and toxicological monitoring, as well as routine pharmaceutical quality control.

## Theory

### Trilinear Model for Second-Order Calibration

In the case of HPLC-DAD analysis, a three-way data array **X**, with dimensions of *I* × *J* × *K* (*I* is the number of elution time scan point, *J* is the number of selected UV spectrum channels, and *K* is the number of samples including calibration and prediction samples) can be produced by stacking a series of HPLC-DAD data obtained for each of the *K* samples. The trilinear component model is expressed in the following form:

1 $$ x_{ijk} = \sum _{n = 1}^{N} a_{in} b_{jn} c_{kn} + e_{ijk} \;\left( {i = 1,2, \ldots ,I;\;j = 1,2, \ldots ,J;\;k = 1,2, \ldots ,K} \right) $$

where *N* denotes the total number of detectable components of interest and the background as well as unknown interferences. *x*_*ijk*_ represents the response intensity of sample k at elution time i and UV spectrum channel j. *c*_*kn*_ is the element (*k, n*) of an *K* × *N* matrix **C** with relative concentrations of the *N* species in *K* samples. *a*_*in*_ is the element (*i*, *n*) of an *I* × *N* matrix **A** with elution profiles of the *N* species. *b*_*jn*_ is the element (*j*, *n*) of an *J* × *N* matrix **B** with spectral profiles of the *N* species, and *e*_*ijk*_ is the element of the three-way residual array **E** (*I* × *J* × *K*).

### ATLD Algorithm

The ATLD algorithm was developed by utilizing the alternating least-squares principle to solve the trilinear model proposed by Wu et al. [47]. Moore–Penrose generalized the inverse based on singular value decomposition and alternating iterative strategy to improve the performance of trilinear decomposition; the loss function reaches a minimum because of developed insensitivity to excessive component numbers, thus resulting in improved convergence. Moreover, with an appropriate signal-to-noise ratio, ATLD yields reasonable results even with high data collinearity. ATLD alternately minimizes the objective functions (2), (3), and (4) to update the qualitative profiles (A and B) and the relative concentrations (C) of individual components:

2 $$ \sigma_{1} \left( {\mathbf{C}} \right) = \mathop \sum \limits_{k = 1}^{K} \left\| {X_{..k} - {\mathbf{A}}diag\left( {\varvec{c}_{(k)} } \right){\mathbf{B}}^{T} } \right\|_{F}^{2} $$

3 $$ \sigma_{2} \left( {\mathbf{A}} \right) = \mathop \sum \limits_{i = 1}^{I} \left\| {X_{i..} - {\mathbf{B}}diag\left( {\varvec{a}_{(i)} } \right){\mathbf{C}}^{T} } \right\|_{F}^{2} $$

4 $$ \sigma_{3} \left( {\mathbf{B}} \right) = \mathop \sum \limits_{j = 1}^{J} \left\| {X_{.j.} - {\mathbf{C}}diag\left( {\varvec{b}_{(j)} } \right){\mathbf{A}}^{T} } \right\|_{F}^{2} $$

### Figures of Merit

FOMs, including SEN, SEL, LOD, and LOQ, are frequently used to optimize analytical methodology and verify the accuracy of the predicted results. In second-order calibration, FOM evaluation is closely related to the calculation of the net analyte signal (NAS), which is defined as the part of the signal that relates uniquely to the NAS. SEN is estimated as the NAS at unit concentration, which is defined as the slope of the calibration curve in the context of univariate calibration. SEL is the ratio between SEN and the total signal. The LOD of a method is the lowest quantity of a substance that can be distinguished from its absence (blank value) within a stated confidence limit, and the LOQ of a method is the limit at which the difference between two different values can be determined. The formulas of FOMs are as follows:

5 $$ {\text{SEN}} = \lambda \left\{ {\left[ {({\mathbf{A}}^{T} {\mathbf{A}})^{ - 1} } \right] \times \left[ {({\mathbf{B}}^{T} {\mathbf{B}})^{ - 1} } \right]} \right\}_{nn}^{ - 1/2} $$

6 $$ {\text{SEL}} = \left\{ {\left[ {\left( {{\mathbf{A}}^{T} {\mathbf{A}}} \right)^{ - 1} } \right] \times \left[ {\left( {{\mathbf{B}}^{T} {\mathbf{B}}} \right)^{ - 1/2} } \right]} \right\}_{nn}^{ - 1/2} $$

7 $$ {\text{LOD}} = 3.3s(0) $$

8 $$ {\text{LOQ}} = 10s(0) $$

where *nn* means the (*n*, *n*) diagonal element of the matrix[(**A** ^*T*^ **A**)^−1^ × (**B** ^*T*^ **B**)^−1^], λ is the total signal for component *n* at unit concentration, and the symbol × indicates the Hadamard product. s(0) is the standard deviation in the predicted concentration for three different background blank samples, in the algorithms.

RMSE can be calculated using the formula as ${\text{RMSEP}} = \left[ {\frac{I}{I - 1}\mathop \sum \limits_{i = 1}^{I} (C_{act} - C_{pred} )^{2} } \right]^{1/2}$, where *I* is the number of prediction samples, *C*_*act*_ and *C*_*pred*_ are the actual and predicted concentrations of the analytes, respectively.

## Materials and Methods

### Chemicals and Reagents

HPLC-grade methanol was purchased from Tedia (USA). Acetic acid (analytical grade) was obtained from Sinopharm Chemical Reagent Co., Ltd. (Shanghai, China). Standards of gentiopicroside, loganic acid, swertiamarin and sweroside were provided by the National Institutes for Food and Drug Control (China). Healthy adult healthy male Sprague–Dawley (SD) rats were purchased from Hubei Research Center of Experimental Animals (SCXK(E)2008-0005).

### Instrument

HPLC was performed using an UltiMate 3000 liquid chromatographic system (Thermo-Dionex Corporation, USA) equipped with a DAD, an auto sampler, and a column compartment. Separation was carried out in a C_18_ column (250 × 4.6 mm, 5.0 mm particle size, Thermo Scientific Syncronis, USA). A centrifuge (Star Scientific Instrument Co., Ltd., China) and ultrasonic instrument (China) were used during sample preparation.

Mass spectrometry was conducted on an Agilent 6520 Q-TOF tandem mass spectrometer equipped with an electrospray ionization (ESI) source (Agilent Corp., USA).

### Sample Preparation

#### Preparation of the Calibration and Validation Samples

Stock standard solutions of GPS, LOG, SWM, and SWS were prepared separately in methanol at concentrations of 1.56, 2.08, 1.68, and 1.20 mg mL^−1^. The first fourteenth Samples (C1–C14) were built as a calibration set. In addition, 10 samples (V1–V10) as a test set were prepared with the analytes concentrations within its corresponding calibration range, which were used to validate the chemometric model. The concentrations of four analytes in both calibration and validation samples were listed in Table 1.

**Table 1:** Concentration of each analyte in calibration and validation samples

Samples	Spiked value (µg mL^−1^)			
	GPS	LOG	SWM	SWS
Calibration samples				
C1	34.63	0.00	0.00	0.00
C2	0.00	45.76	0.00	0.00
C3	0.00	0.00	44.35	0.00
C4	0.00	0.00	0.00	42.00
C5	4.99	41.60	6.05	38.16
C6	8.11	37.44	10.08	34.32
C7	11.23	33.28	14.11	30.48
C8	14.35	29.12	18.14	26.64
C9	17.47	24.96	22.18	22.80
C10	20.59	20.80	26.21	18.96
C11	23.71	16.64	30.24	15.12
C12	26.83	12.48	34.27	11.28
C13	29.95	8.32	38.30	7.44
C14	33.07	4.16	42.34	3.60
Validation samples				
V1	3.43	43.68	4.03	40.08
V2	6.55	39.52	8.06	36.24
V3	9.67	35.36	12.1	32.4
V4	12.79	31.2	16.13	28.56
V5	15.91	27.04	20.16	24.72
V6	19.03	22.88	24.19	20.88
V7	22.15	18.72	28.22	17.04
V8	25.27	14.56	32.26	13.2
V9	28.39	10.4	36.29	9.36
V10	31.51	6.24	40.32	5.52

#### Pretreatment of Rat Urine and Feces Samples

Protein precipitation was applied to extract four bioactive ingredients from rat urine and feces samples. Five healthy male SD rats were housed in stainless-steel metabolic cages equipped with urine–feces separators. Urine and feces samples were collected at different time intervals (namely, 0–2, 2–4, 4–8, 8–12, 12–24, 24–36, and 36–48 h) post-dosage. Feces samples collected from different time points were dried using a heated oven at 40 °C and then pulverized. 5 mL g^−1^ physiological saline solution was added and homogenized with the pulverized feces, and 100 μL feces supernatant from homogenate was blended with 400 μL of methanol. The mixture was vigorously vortexed for approximately 1 min and then centrifuged at 12000 rpm for 15 min at 4 °C. Then, 400 μL of the supernatant was transferred to a new 1.5 mL centrifuge tube and completely evaporated under a gentle stream of nitrogen. Residues were dissolved in 100 μL of the mobile phase, and 10 μL of sample solutions was injected into the HPLC-DAD for analysis. Urine samples were treated in a similar manner; however, 500 µL methanol was used in the protein precipitation stage, and 500 µL supernatant was dried.

#### Preparation of Quality Control Samples in Urine and Feces

Three levels of high, middle, and low concentration were prepared as working solution by diluting the stock standard solutions with methanol. GPS, LOG, SWM, and SWS in working solutions with high concentration level were at 49.92, 58.24, 33.60, and 24.00 μg mL^−1^, respectively. GPS, LOG, SWM, and SWS in working solutions with level of middle concentration were at 30.58, 36.40, 23.86, and 18.6 μg mL^−1^, respectively. GPS, LOG, SWM, and SWS in working solutions with low concentration were at 11.23, 14.56, 14.11, and 13.2 μg mL^−1^, respectively. For urine quality control (QC) samples; high, middle and low concentration levels of working solution (60 µL) were transferred separately into three 1.5 mL centrifuge tubes and dried with nitrogen, and then 300 μL blank urine samples was added to each tube. Methanol (1500 µL) was used for protein precipitation. The mixture was vigorously vortexed for approximately 1 min and then centrifuged at 12000 rpm and 4 °C for 15 min. Then, 1500 μL of the supernatant was transferred to a new 1.5 mL centrifuge tube and evaporated to dryness under a gentle stream of nitrogen. Residues were dissolved in 300 μL of mobile phase before analysis. Feces QC samples were treated in a similar manner, except 1200 µL methanol was used in the protein precipitation stage, and 1200 µL supernatant was dried. The concentrations of the four analytes in urine and feces QC samples were summarized in Table 2.

**Table 2:** Concentrations of four analytes in urine and feces QC samples

Analytes	GPS (µg mL^−1^)		LOG (µg mL^−1^)		SWM (µg mL^−1^)		SWS (µg mL^−1^)	
	Urine	Feces	Urine	Feces	Urine	Feces	Urine	Feces
H	8.32	7.99	9.71	9.32	5.60	5.38	4.00	3.84
M	5.10	4.89	6.07	5.82	3.98	3.82	3.10	2.98
L	1.87	1.80	2.43	2.33	2.35	2.26	2.20	2.11

#### Preparation of *Radix Gentianae Macrophyllae* Water Decoction

One hundred grams of *Radix Gentianae Macrophyllae* was mixed with 1000 mL distilled water and heated to boiling for 30 min. The residual part was added in 1000 mL distilled water and heated to boiling for 30 min. This procedure was repeated twice. Eventually, 50 mL water extract was obtained at a concentration of 2 g mL^−1^ crude drug. The water extract was diluted separately to obtain concentrations of 1 and 1.5 g mL^−1^ crude drug for storage at 4 °C.

### Excretion Study

For urinary and fecal excretion study, 5 healthy SD male rats were housed in stainless-steel metabolic cages with free access to water and fasted for 12 h before the experiment. Urine and feces samples of each rat at 0 h (control) were collected before the experiment, and a *Radix Gentianae Macrophyllae* decoction at a single dose of 16 g kg^−1^ was administered as described above. Food was returned at approximately 4 h post-dosing, and then urine and feces were collected at 0–2, 2–4, 4–8, 8–12, 12–24, 24–36, and 36–48 h post-dosing as prediction samples. Urine volume was recorded, and dried feces was weighed. Then, specimens were stored at −20 °C until analysis.

### Establishment of Detection Conditions

Liquid chromatography was performed on liquid chromatographic system (DIONEX Corporation, USA) equipped with DAD. Separation was carried out on a C_18_ column (Thermo Scientific Syncronis, 250 × 4.6 mm, 5.0 mm particle size). The isocratic mobile phase consisted of methanol and water acidified with 0.1% acetic acid solution (3:7, v/v) and was pumped at a flow rate of 1.0 mL min^−1^ with 10 μL injection volume. Column temperature was set at 30 °C. Photometric detection was performed in the range of 190–600 nm with a spectral interval of 1 nm.

Mass spectra were detected on an Agilent 6520 Q-TOF tandem mass spectrometer equipped with an ESI source (Agilent Corp., USA). The LOG ESI source was set to negative ionization mode, whereas the ESI source of GPS, SWM, and SWS were set to positive ionization mode. The MS operating conditions were optimized as follows: scanning spectrum range from 100 to 800 m/z, nebulizer pressure of 30 psi (N_2_), dry gas temperature of 300 °C, spray voltage of 3500 V, skimmer voltage of 125 V, and nitrogen at 10 L min^−1^ as dry gas.

Three-way data produced by HPLC-DAD were imported to a microcomputer with a Windows Server 2008 operating system and analyzed in Matlab environment.

## Results and Discussion

### Establishment and Validation of Calibration Models for Excretion Study

**Fig. 1:**
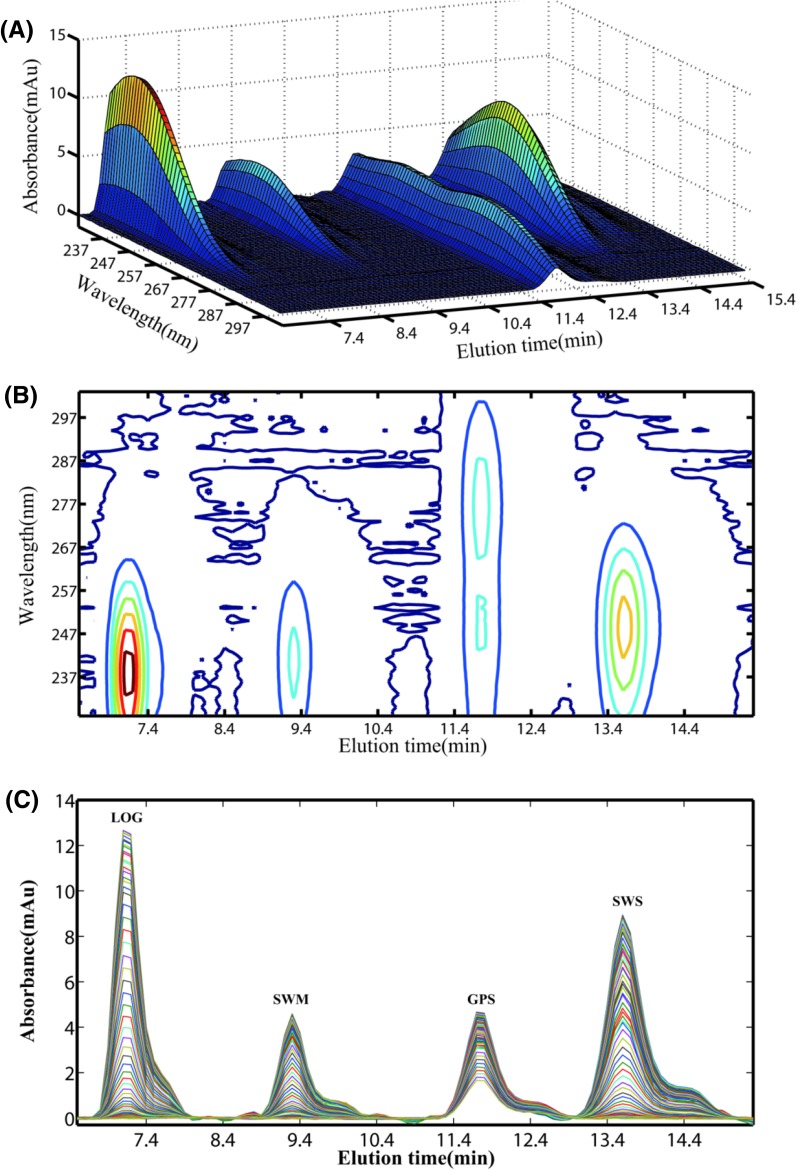
**a** Three-dimensional plot of HPLC-DAD data for four analytes; **b** contour plot of HPLC-DAD data for four analytes; **c** chromatograms of four analytes at different wavelengths

Four bioactive ingredients were eluted within 16 min in isocratic mode, as shown in Fig. 1. The four analytes were separated successfully under current chromatographic separation condition.

**Fig. 2:**
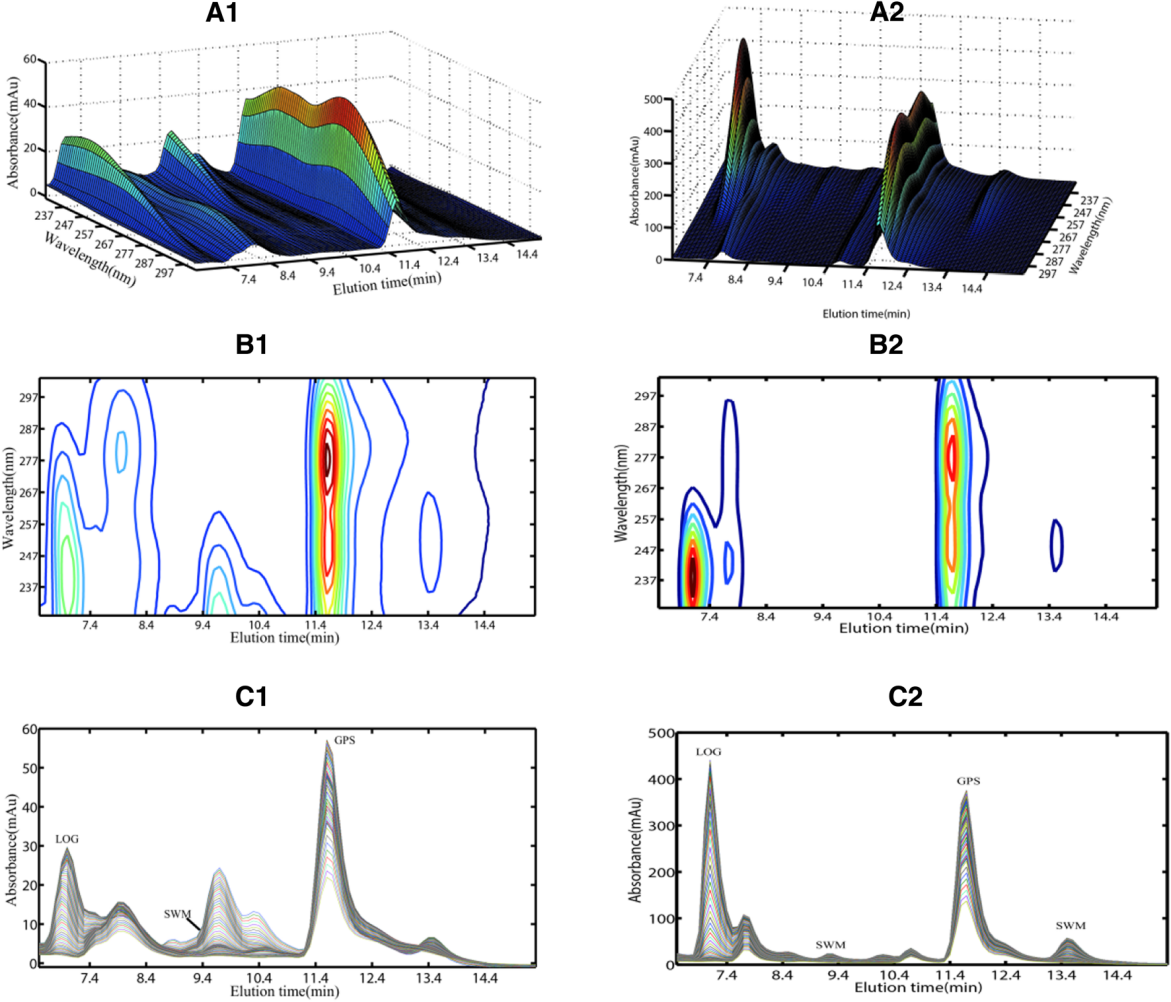
Three-dimensional plot of a typical chromatogram of urine (**a1**) and feces samples (**a2**) from rat administered with low dosage of *Radix Gentianae Macrophyllae* water decoction (16 g kg^−1^) within 2–4 h; **b1**, **b2** are contour plots corresponding to **a1**, **a2**, respectively. Chromatograms recorded at various wavelength channels for urine (**c1**) and feces samples (**c2**)

The rat urine and feces samples were administered with alow-level *Radix Gentianae Macrophyllae* water decoction (16 g kg^−1^) within 2–4 h to show the typical chromatographic plot in Fig. 2. Unfortunately, chromatographic profiles from four bioactive ingredients heavily overlapped with that of biological matrix interferences from urine and feces samples under the same chromatographic separation condition. Therefore, traditional HPLC analytical methods provide invalid quantitative results without careful extraction and separation procedures. Great effort may be necessary to optimize the chromatographic conditions of the separation for the problem, but a complicated chromatographic condition means more time and resources would be consumed. Alternatively, one can resort to the second-order calibration method based on ATLD algorithm, which allows the relative chromatographic, spectral, and concentration profiles of analytes to be extracted even in the presence of uncalibrated interferences.

Therefore, for excretion studies, prior to an estimate of the concentration of four bioactive ingredients in urine and feces, Validation samples (V1–V10) were utilized to validate whether the calibration model was reliable. In order to avoid the interference from irrelevant data and simplify the analytical process, total chromatographic data registered for each sample was segmented in two three-way data array for mathematical modeling, namely the first data array (89 × 76 × 24) was constructed by an elution time range of 6.5–15.3 min (Δt = 0.1 s) and a wavelength range of 225–300 nm (Δλ = 1 nm) for the analysis GPS, LOG and SWS, the second data array (23 × 49 × 24) was constructed by selecting the elution time range of 8.4–10.6 min (Δt = 0.1 s) and the wavelength range of 232–280 nm (Δλ = 1 nm) for the analysis of SWM. The quantitative results of concentrations of validation samples (V1–V10) were shown in Table 3.

**Table 3:** Predicted concentrations of validation samples

Validation sample	Predicted value (µg mL^−1^) [recovery (%)]			
	GPS	LOG	SWM	SWS
V1	3.27 [95.3]	44.82 [102.6]	4.03 [100.0]	42.49 [106.0]
V2	6.47 [98.8]	39.86 [100.9]	7.94 [98.5]	36.69 [101.2]
V3	9.51 [98.4]	35.3 [99.8]	12.06 [99.7]	32.27 [99.6]
V4	12.83 [100.3]	30.78 [98.7]	16.15 [100.1]	28.49 [99.8]
V5	15.88 [99.8]	26.79 [99.1]	20.06 [99.5]	24.27 [98.2]
V6	19.89 [104.5]	23.49 [102.7]	25.11 [103.8]	21.47 [102.8]
V7	22.40 [101.1]]	18.43 [98.5]	28.96 [102.6]	16.67 [97.8]
V8	25.78 [102.0]	14.39 [98.8]	32.82 [101.7]	13.02 [98.6]
V9	28.48 [100.3]	10.44 [100.4]	36.15 [99.6]	9.17 [98.0]
V10	31.85 [101.1]	5.79 [92.8]	41 [101.7]	5.64 [102.2]
Average recovery (%)	100.2 ± 2.4	99.4 ± 2.8	100.7 ± 1.7	100.4 ± 2.6
RMSEP	1.51	2.30	1.90	2.41
*T* (*t*-test)	0.21 < ${\text{t}}_{0.025}^{9}$	0.66 < ${\text{t}}_{0.025}^{9}$	1.39 < ${\text{t}}_{0.025}^{9}$	0.52 < ${\text{t}}_{0.025}^{9}$

The average recoveries of GPS, LOG, SWS, and SWM in 10 validation samples were 100.2 ± 2.4, 99.4 ± 2.8, 100.4 ± 2.6, and 100.7 ± 1.7%, and the calculated RMSEP values were 1.51, 2.30, 2.41 and 1.90 μg mL^−1^, respectively. The *t*-test was carried out to compare recoveries with the ideal value of 100% for all four analytes. $T < t_{0.025}^{9}$, suggesting no significant difference between the results under the confidence level of 95%. The results clearly indicate that ATLD are reliable for the simultaneous quantification of GPS, LOG, SWM, and SWS. In addition, the QC samples in urine and feces were adopted to investigate extraction recovery, precision, and stability of the established method. The QC samples were analyzed in triplicate in a day. This assay was repeated for 3 days. Satisfactory extraction capacities toward the four analytes can be obtained and the extraction recovery were range from 74.1 ± 1.1 to 107.4 ± 3.7%. The corresponding results of intra-day and inter-day accuracy and precision were listed in Table 4. Apparently, both Intra- and inter-day relative standard deviation of the concentrations of four analytes in urine and feces QC samples for middle and high concentrations were within ± 10%, for low concentrations were in the range of ± 20%. Thus, this method is accurate and precise for the direct determination of four bioactive ingredients in urine and feces samples.

**Table 4:** Intra-day and inter-day accuracy and precision of four bioactive ingredients in rat urine and feces QC samples

Matrix	Analytes	Concentration (μg mL^−1^)		Precision (RSD, %)		Accuracy (RE, %)	
		Spiked	Measured (mean ± SD)	Intra-day	Inter-day	Intra-day	Inter-day
Urine	GPS	8.32	8.44 ± 0.13	1.5	6.3	1.4	− 2.5
		5.10	5.19 ± 0.24	4.7	4.6	1.8	− 1.2
		1.87	2.21 ± 0.50	14.9	14.4	− 8.1	6.6
	LOG	9.71	10.9 ± 0.07	0.7	2.9	3.9	0.7
		6.07	6.84 ± 0.17	2.4	7.4	12.8	− 3.3
		2.43	1.77 ± 0.13	4.6	16.2	7.7	4.2
	SWM	5.60	4.77 ± 0.25	5.2	6.7	− 14.9	− 6.5
		3.98	3.11 ± 0.16	5.0	3.1	− 15.1	− 6.9
		2.35	2.54 ± 0.39	15.5	9.5	7.9	− 7.8
	SWS	4.00	3.86 ± 0.84	3.9	1.3	− 12.4	5.5
		3.10	3.28 ± 0.04	1.1	10.0	5.8	2.5
		2.20	2.49 ± 0.15	5.8	17.6	13.3	− 7.3
Feces	GPS	7.99	8.04 ± 0.39	5.2	4.9	1.3	0.6
		4.89	4.80 ± 0.12	2.4	2.5	− 0.4	− 2.0
		1.80	1.70 ± 0.04	4.8	2.6	1.2	− 5.4
	LOG	9.32	9.38 ± 0.08	0.8	6.8	0.6	2.9
		5.82	5.77 ± 0.02	0.3	3.6	− 0.9	2.4
		2.33	2.26 ± 0.01	0.7	6.1	− 3.2	8.3
	SWM	5.38	5.02 ± 0.36	7.3	3.0	− 6.6	− 0.6
		3.82	3.38 ± 0.07	2.1	4.9	− 11.4	− 1.8
		2.26	2.00 ± 0.09	4.7	7.7	− 11.2	− 0.6
	SWS	3.84	4.06 ± 0.21	2.0	3.6	5.8	− 0.3
		2.98	2.50 ± 0.09	12.3	13.6	− 4.5	− 2.3
		2.11	1.92 ± 0.03	1.4	9.1	− 9.1	− 3.5

Moreover, the stability of the analytes in rat urine and feces QC samples were determined. Four different sample preprocessing methods, including short term, long term, freeze–thaw, and post-preparation, were employed. Short-term and long-term stability were evaluated by storing the frozen samples at room temperature for 8 h and at − 80 °C in a freezer for 21 days. Freeze–thaw stability was assessed after three cycles; then, QC samples were frozen at − 80 °C for 12 h and thawed. Post-preparation was evaluated after the processed samples were stored in an auto-sampler tray for 24 h. The high, middle, and low concentration levels of QC samples were predicted in triplicate with the aid of ATLD and then compared with actual concentrations. All the relative deviations were within ± 15%. The stability of QC samples was acceptable under indicated storage conditions, as shown in Table 5. Hence, this method provides satisfactory stability for determining the four analytes and offer considerable potential to be tailored as a routine method for analyzing the excretion of TCM.

**Table 5:** Stability of four analytes in urine and feces samples based on ATLD algorithm

Matrix	Analytes	Concentration	Room-temperature for 8 h		In the auto-sampler for 24 h		Three freeze cycles in rat urine		Long-term stability (− 80 °C, 21 days)	
		Spiked (μg mL^−1^)	Measured (mean ± SD)	RE (%)	Measured (mean ± SD)	RE (%)	Measured (mean ± SD)	RE (%)	Measured (mean ± SD)	RE (%)
Urine	GPS	8.32	7.46 ± 0.18	− 10.2	8.39 ± 0.17	1.0	9.17 ± 0.78	10.4	7.79 ± 0.34	− 6.2
		5.10	4.99 ± 0.16	− 2.1	4.64 ± 0.06	− 8.7	5.03 ± 0.09	− 1.2	4.83 ± 0.12	− 5.1
		1.87	1.36 ± 0.16	− 14.9	1.91 ± 0.06	2.1	2.01 ± 0.36	7.8	1.94 ± 0.13	3.7
	LOG	9.71	9.91 ± 0.05	2.2	10.23 ± 0.05	5.6	9.27 ± 0.07	− 4.3	8.36 ± 0.16	− 13.8
		6.07	6.71 ± 0.04	10.8	6.27 ± 0.04	3.6	5.85 ± 0.11	− 3.4	6.13 ± 0.17	1.3
		2.43	2.41 ± 0.09	− 0.5	2.79 ± 0.05	15.0	2.75 ± 0.10	13.5	2.08 ± 0.05	− 14.2
	SWM	5.60	5.53 ± 0.35	− 1.1	5.30 ± 0.01	− 5.3	5.17 ± 0.08	− 7.6	5.18 ± 0.91	− 7.5
		3.98	4.13 ± 0.27	3.9	4.46 ± 0.07	12.4	4.25 ± 0.05	7.1	4.47 ± 0.18	12.6
		2.35	2.40 ± 0.13	2.1	2.34 ± 0.12	− 0.2	2.41 ± 0.02	2.4	2.33 ± 0.34	− 0.7
	SWS	4.00	4.04 ± 0.57	1.1	3.91 ± 0.18	− 2.2	4.10 ± 0.66	2.8	3.87 ± 0.88	− 3.1
		3.10	3.49 ± 0.36	13.4	3.28 ± 0.28	5.9	2.89 ± 0.15	− 6.8	3.18 ± 0.36	2.8
		2.20	2.49 ± 0.07	2.1	2.50 ± 0.07	13.5	1.97 ± 0.08	− 10.3	2.01 ± 0.02	− 8.6
	GPS	7.99	8.66 ± 0.31	8.5	8.63 ± 0.10	8.1	7.11 ± 0.67	− 11.0	7.26 ± 0.79	− 9.1
Feces		4.89	4.80 ± 0.14	− 2.0	4.96 ± 0.05	1.4	4.18 ± 0.30	− 14.6	4.76 ± 0.06	11.0
		1.80	1.66 ± 0.06	− 7.4	2.10 ± 0.03	16.7	1.69 ± 0.32	− 5.6	1.72 ± 0.29	− 4.4
	LOG	9.32	9.73 ± 0.04	4.5	9.63 ± 0.13	3.4	8.55 ± 0.69	− 8.3	10.01 ± 0.16	7.4
		5.82	6.40 ± 0.10	9.8	6.43 ± 0.19	− 3.9	5.54 ± 0.14	− 4.9	6.18 ± 0.17	6.2
		2.33	2.92 ± 0.61	− 5.0	2.44 ± 0.04	4.7	2.20 ± 0.03	− 5.5	2.15 ± 0.02	− 7.9
	SWM	5.38	6.16 ± 0.45	14.6	5.55 ± 0.48	3.2	5.32 ± 0.47	− 1.0	4.99 ± 0.57	− 7.1
		3.82	3.86 ± 0.36	1.2	3.71 ± 0.23	− 2.8	4.12 ± 0.32	8.0	3.40 ± 0.21	− 11.0
		2.26	2.19 ± 0.04	− 3.0	2.44 ± 0.16	8.1	2.46 ± 0.01	9.0	2.59 ± 0.18	14.6
	SWS	3.84	4.36 ± 0.27	13.5	4.01 ± 0.05	4.4	3.95 ± 0.38	2.9	3.64 ± 0.06	− 5.2
		2.98	3.11 ± 0.09	4.6	3.05 ± 0.02	2.6	3.00 ± 0.19	0.8	2.92 ± 0.18	0.8
		2.11	2.26 ± 0.02	6.8	2.30 ± 0.03	9.1	2.39 ± 0.27	13.0	2.14 ± 0.15	1.1

### Study on Kinetics of Excretion

#### Simultaneous Determination of Four Bioactive Ingredients in Rat Excrement

**Fig. 3:**
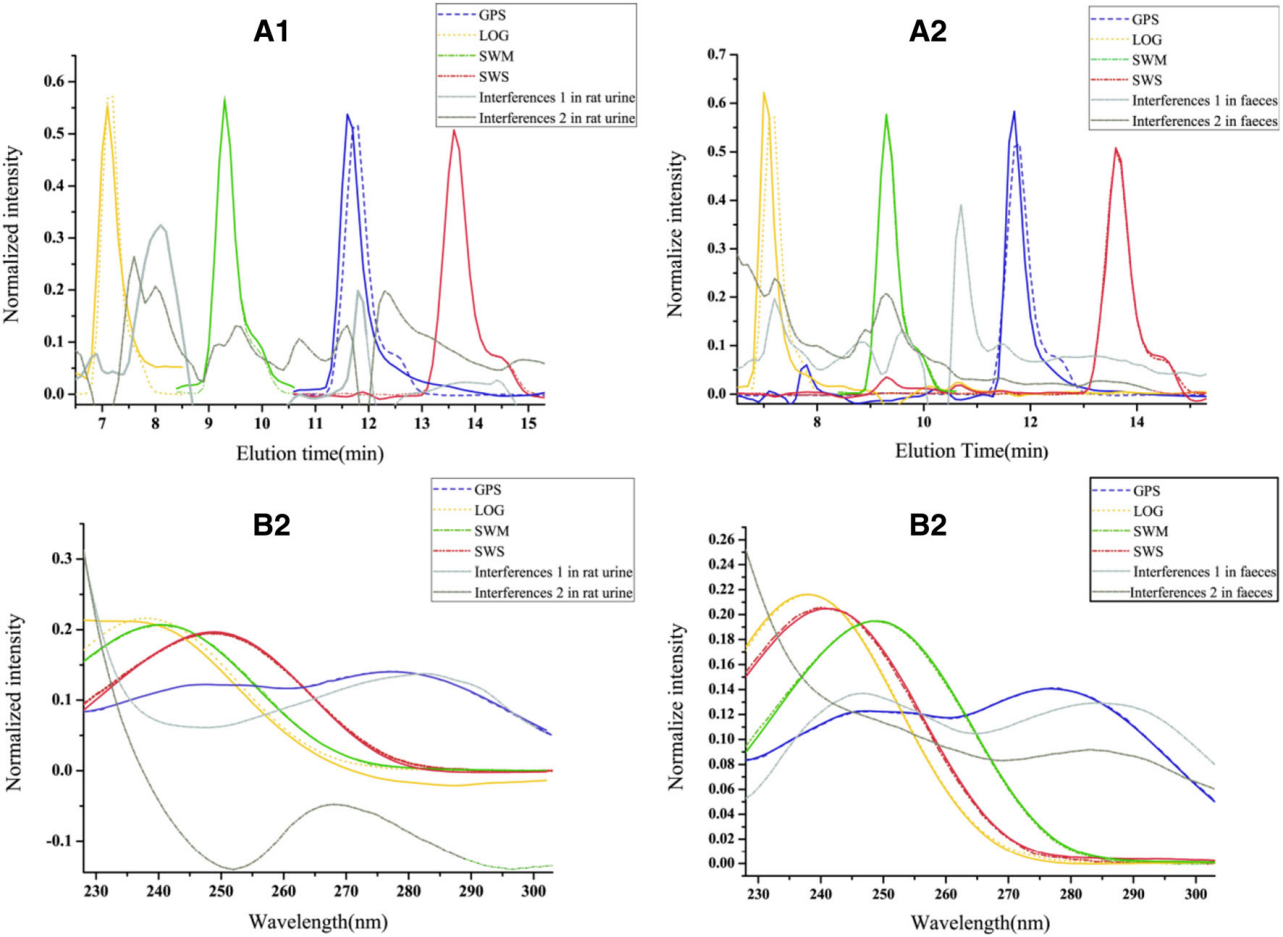
Actual and resolved elution profiles (**a1**) and wavelength profiles (**b1**) of four bioactive ingredients in rat urine by using ATLD. Actual and resolved elution profiles (**a2**) and wavelength profiles (**b2**) of four bioactive ingredients in rat feces by using ATLD. Solid lines, dotted solid lines, and dotted lines represent the actual spectral profiles of four analytes, the loadings for four analytes, and inherent interference from rat urine or feces, respectively

The established method based on second-order calibration strategy was applied to analyze urine and feces samples after the oral administration of *Radix Gentianae Macrophyllae* water decoctions. After the appropriate component numbers were estimated using core consistency diagnostic (CORCONDIA) [48], three-way data arrays obtained from HPLC-DAD analysis for calibration and prediction samples were decomposed using ATLD. The actual spectral and elution time profiles together with their corresponding loadings were obtained from the decomposition of the HPLC-DAD data array by ATLD. Resolution results for rat urine and feces samples administered with *Radix Gentianae Macrophyllae* water decoction at a low concentration level are shown in Fig. 3. Loadings in the wavelength and elution time modes of SWM and SWS were almost similar to the actual values, implying the reliability and stability of the new analytical strategy. The slight deviation between the actual and fitting chromatographic profiles of LOG, GPS, and consistency degree (CD) values was compared to assess the consistency of the resolution and actual chromatographic profiles. The formula of CD is expressed as: CD = min (cos (*a*_a_, *a*_r_), cos (*b*_a_, *b*_r_)), where cos means cosine, *a*_a_ and *b*_a_ are the actual chromatographic and spectral profiles of the target analyte, respectively, and *a*_r_ and *b*_r_ are the resolved chromatographic and spectral profiles, respectively. The CD values are close to 1, indicating highly effective mathematical separation of bioactive ingredient information in biological excretion samples. The CDs of GPS and LOG in chromatographic profiles for urine samples were calculated as 0.8111 and 0.8053, respectively, and CDs of GPS and LOG in spectral profiles for urine samples were 0.9831 and 0.9824, respectively. The requirement of quantitative analysis was fulfilled, and satisfactory results could be obtained for feces samples by using a similar calculation method. These results are acceptable in practical applications and further confirm that the proposed method accurately quantifies the analytes of interest even in different complex matrices.

**Fig. 4:**
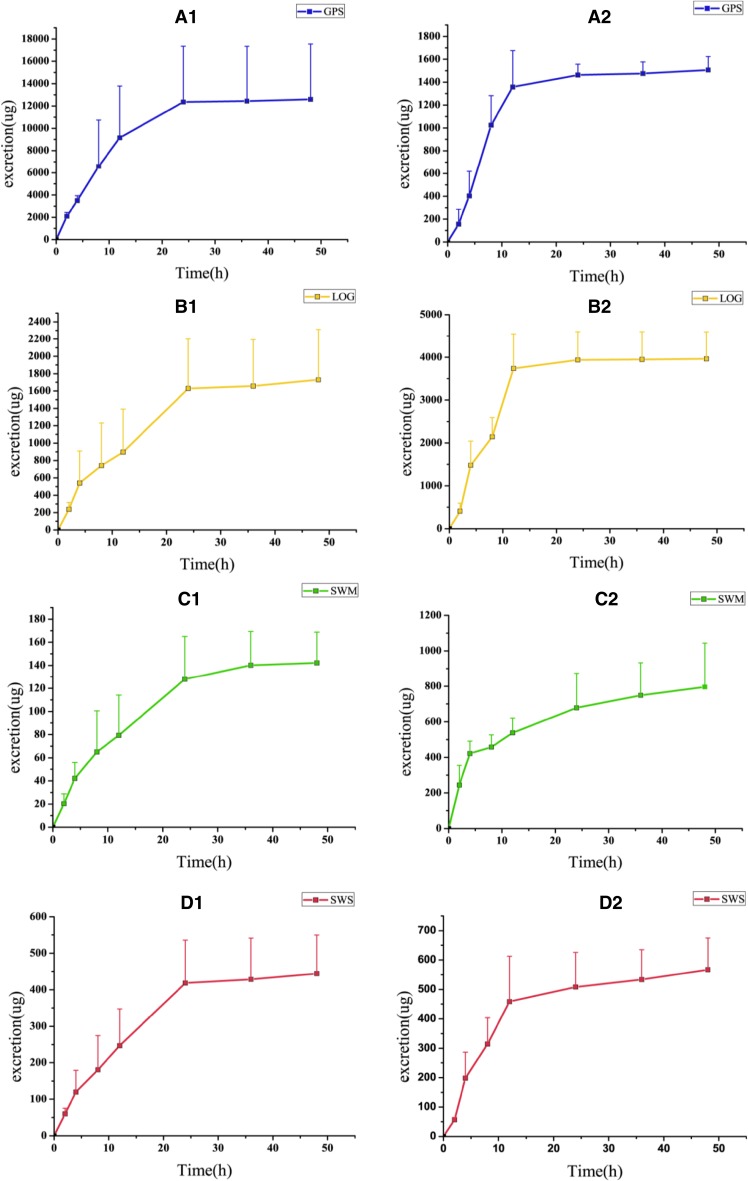
The cumulative excretion(mean + SD)of four bioactive ingredients in urine (**a1**, **b1**, **c1**, **d1**) and in feces (**a2**, **b2**, **c2**, **d2**), respectively

The resolved relative concentration contributions for each analyte of interest were independently regressed against the corresponding standard concentrations from calibration samples. Thus, we can predict the concentrations of the four active ingredients in the excrement of rats orally administered with low, middle, and high dosages at different time points. The synergistic relationships and cumulative excretion of the four bioactive components of *Radix Gentianae Macrophyllae* are shown in Fig. 4. The maximum excretion for GPS, LOG, and SWS was recorded in the time range of 4–8 h, whereas the corresponding data for SWM were recorded within 0–2 h. In urine samples, the cumulative excretion values of GPS, LOG, SWM, and SWS within 0–48 h were only 7.5, 4.7, 6.3, and 9.4% of the initial dosage, respectively; hence, these components are limitedly excreted in their initial forms. In feces samples, the cumulative excretion values of GPS, LOG, SWM, and SWS within 0–48 h were 0.9, 10.6, 35.4, and 12.0% of the initial dosage, respectively, with SWM exhibiting higher fecal excretion compared with other bioactive components.

According to the available literature on the pharmacokinetics of GPS, SWM, and SWS, the three components show low oral bioavailability of 39.6, 10.0, and 0.31%, respectively. The four active components of *Radix Gentianae Macrophyllae* may be converted into other components via biotransformation, and GPS reportedly transform into five active constituents [49]. In addition, SWS exhibits high biliary excretion rate of approximately 31.2% with low bioavailability in vivo. This low availability may be due to the high polarity of SWS, which makes direct absorption difficult. Otherwise, SWS is metabolized by intestinal bacteria by first-pass effect. GPS, SWM, and SWS are mainly excreted by the kidneys and in bile and feces. Based on these considerations, the present study on the pharmacokinetics of *Radix Gentianae Macrophyllae* is of considerable significance in pharmacokinetic research.

#### Figures of Merit

FOMs, including SEL, SEN, LOD, and LOQ, should be calculated to validate the second-order calibration method. FOMs in the matrix of rat urine and feces samples are listed in Table 6. Results show that the new method can provide satisfactory predictive results in the quantitative analysis of four analytes in rat excrement, overcome the influences of unknown interferences from complicated biological matrix, and avoid tedious pretreatments, such as solid-phase extraction. Therefore, experimental efficiency and accuracy can be markedly improved.

**Table 6:** FOM of four analytes in urine and feces samples using ATLD algorithm

	n	Urine samples				Feces samples			
		LOD (μg mL^−1^)	LOQ (μg mL^−1^)	SEN (mL μg^−1^)	SEL	LOD (μg mL^−1^)	LOQ (μg mL^−1^)	SEN (mL μg^−1)^	SEL
GPS	1	0.02	0.05	2.45	0.67	0.06	0.18	1.93	0.56
	2	0.59	1.80	3.24	0.57	0.30	0.90	0.81	0.22
	3	0.59	1.80	2.23	0.47	0.06	0.18	1.93	0.55
	4	0.06	0.18	1.44	0.47	0.06	0.18	1.79	0.58
	5	0.06	0.18	1.49	0.49	0.06	0.18	1.28	0.38
LOG	1	0.43	1.30	2.63	0.62	0.48	1.44	1.09	0.29
	2	0.16	0.48	2.01	0.47	0.16	0.48	0.78	0.21
	3	0.42	1.27	1.35	0.35	0.08	0.24	1.81	0.52
	4	0.02	0.07	2.34	0.57	0.87	2.64	1.05	0.28
	5	0.32	0.96	1.01	0.39	0.08	0.24	0.98	0.27
SWM	1	0.07	0.21	0.18	1.55	0.70	2.11	0.91	0.46
	2	0.38	1.16	0.57	0.58	0.97	2.95	0.93	0.42
	3	0.40	1.22	0.13	0.34	0.15	0.45	0.68	0.37
	4	0.26	0.78	0.62	0.42	0.17	0.52	0.69	1.00
	5	0.15	0.47	0.40	0.39	0.64	1.94	0.73	0.43
SWS	1	0.13	0.39	1.03	0.26	0.04	0.11	0.86	0.25
	2	0.08	0.24	1.32	0.51	0.36	1.09	0.84	0.28
	3	0.30	0.90	1.66	0.57	0.23	0.69	0.78	0.26
	4	0.28	0.86	1.53	0.50	0.00	0.01	0.87	0.25
	5	0.27	0.82	1.86	0.64	0.09	0.26	0.81	0.23

### Verification of HPLC-MS Results

For verifying the accuracy of the simultaneous determination of four analytes, urine samples collected from 2 to 4 and 4 to 8 h after the oral administration of a 16 g kg^−1^ *Radix Gentianae Macrophyllae* water decoction were selected as a typical example because of their complex background matrix, which severely overlapped with target analytes. Student’s *t*-test was applied to compare the significant difference of concentrations based on ATLD and HPLC-MS. The simultaneous determination of the four bioactive ingredients in urine samples by ATLD and LC-MS is compared in Table 7. The RE values of four bioactive components was less than 16%, and the predicted concentrations by ATLD and the concentrations provided by HPLC-MS were not significantly different at 95% confidence level This result indicates that ATLD can directly determine the concentration of four bioactive ingredients in complex urine and feces samples.

**Table 7:** The comparison of simultaneous determination of four bioactive ingredients in urine samples by the algorithm of ATLD and LC-MS

Ingredients	t (2–4 h)			t (4–8 h)		
	ATLD (μg mL^−1^)	LC-MS (μg mL^−1^)	RE (%)	ATLD (μg mL^−1^)	LC-MS (μg mL^−1^)	RE (%)
GPS	84.56	84.88	− 0.4	97.36	96.95	0.4
LOG	0.77	0.77	0.0	1.34	1.58	− 15.2
SWM	11.07	11.99	− 7.7	22.97	23.39	− 1.8
SWS	2.14	1.85	15.7	3.50	3.28	6.7
T-test	$0.37 < t_{0.025}^{3}$			$0.52 < t_{0.025}^{3}$		

## Conclusion

In this paper, a novel and effective multi-dimensional quantitative characterization model was successfully established to investigate the synergistic excretion and conduct pharmacokinetic analysis of four bioactive ingredients in *Radix Gentianae Macrophyllae* by using HPLC-DAD and ATLD with aid of region selection. The total cumulative excretion of GPS, LOG, SWM, and SWS in urine and feces samples collected within different excretive time intervals after oral administration of the water decoction was calculated to reveal the synergistic relationships. The accuracy of the proposed method was validated using HPLC-MS. FOMs, including SEN, SEL, LOD, and LOQ, were evaluated. All results indicated that the proposed method can not only provide a convenient, rapid, and reliable reference method for the analysis of complex excretion samples but also show marked potential for further tailoring as a general and promising approach to study the pharmacokinetics of TCM and natural products.

## References

[1] Wang Y, Ahmad B, Duan B, Rui Z, Huang L (2016). Chem. Biodivers..

[2] Jia N, Li Y, Wu Y, Xi M, Hur G, Zhang X, Cui J, Sun W, Wen A (2012). J. Ethnopharmacol..

[3] Cao XY, Wang ZZ (2010). Phytochem. Anal..

[4] Nie AZ, Lin ZJ, Wang Y, Zhang B (2017). Chin. Tradit. Herbal Drugs.

[5] Changliao WL, Chien CF, Lin LC, Tsai TH (2012). J. Ethnopharmacol..

[6] Ma TM, Liu F, Wang R, Gao HQ (2017). Chin. Tradit. Herbal Drugs.

[7] Liu Y, Kong JM, Chia LS, Goh NK (2007). Asian J. Chem..

[8] Kong WJ, Zhao YL, Xiao XH, Jin C, Li ZL (2009). Phytomedicine.

[9] Rao Z, Zhang F, Zhang XY, Zhang GQ, Ma YR, Zhou Y, Qin HY, Wu XA, Wei YH (2015). Biomed. Chromatogr..

[10] Cheng YY, Tsai TH (2016). Molecules.

[11] Choi MK, Lee J, Nam SJ, Kang YJ, Han Y, Choi K, Choi YA, Kwon M, Lee D, Song IS (2017). Mar. Drugs.

[12] Figueiredo TCD, Assis DCSD, Menezes LDM, Silva GRD, Lanza IP, Heneinec LGD, Cancadoa SDV (2015). Talanta.

[13] Moghbel N, Ryu BM, Steadman KJ (2015). J. Chromatogr. B.

[14] Shao Y, Zhang W, Tong L, Huang J, Li D, Nie W, Zhu Y, Li Y, Lu T (2017). Biomed. Chromatogr..

[15] Zheng L, Gong Z, Lu Y, Xie Y, Huang Y, Liu Y, Lan YY, Wang AM, Wang YL (2015). J. Chromatogr. B.

[16] Shariati-Rad M, Irandoust M, Niazi F (2016). J. Anal. Chem..

[17] Aimo J, Promancio E, Damiani PC (2016). Anal. Methods.

[18] Zhang XH, Wu HL, Wang JY, Tu DZ, Kang C, Zhao J, Chen Y, Miu XX, Yu RQ (2013). Food Chem..

[19] Yu YJ, Wu HL, Shao SZ, Kang C, Zhao J, Wang Y, Zhu SH, Yu RQ (2011). Talanta.

[20] Ayvaz H, Rodriguez-Saona LE (2015). Food Chem..

[21] Sun YM, Hai-Long WU, Wang JY, Ru-Qin YU (2013). Chem. J. Chin. Univ..

[22] Boucher CL, Courant F, Royer AL, Jeanson S, Lortal S, Dervilly-Pinel G, Thierry A, Bizec BL (2015). Metabolomics.

[23] Yin XL, Wu HL, Gu HW, Zhang XH, Sun YM, Hu Y, Liu L, Rong QM, Yu RQ (2014). J. Chromatogr. A.

[24] Schenone AV, Culzoni MJ, Marsili NR, Goicoechea HC (2013). Food Chem..

[25] Tu DZ, Wu HL, Li YN, Zhang J, Li Y, Nie CC, Zhang XH, Yu RQ (2012). Anal. Methods.

[26] Wang JY, Wu HL, Chen Y, Zhai M, Qing XD, Yu RQ (2013). Talanta.

[27] Qing XD, Wu HL, Li YN, Nie CC, Wang JY, Zhu SH, Yu RQ (2012). Anal. Methods.

[28] Fuentes E, Cid C, Báez ME (2015). Talanta.

[29] Yang R, Zhao N, Xiao X, Yin G, Y S, Liu J, Liu W (2016). Opt. Express.

[30] Vosough M, Mashhadiabbas EH (2013). Talanta.

[31] Pagani AP, Ibañez GA (2014). Talanta.

[32] Sheffield WP, Bhakta V (2016). Biochem. Biophys. Res. Commun..

[33] Yan ECY, Wang Z, Fu L (2015). Phys. Chem. B.

[34] Vosough M, Eshlaghi SN, Zadmard R (2015). Spectrochim. Acta.

[35] Li SS, Wu HL, Liu YJ, Gu HW, Yu RQ (2013). Chin. Chem. Lett..

[36] Pagani AP, Ibanez GA (2014). Talanta.

[37] Sun YM, Wu HL, Wang JY, Liu Z, Zhai M, Yu RQ (2014). J. Chromatogr. B.

[38] Kooshki M, Abdollahi H, Bozorgzadeh S, Haghighi B (2011). Electrochim. Acta.

[39] Piccirilli GN, Escandar GM (2010). Analyst.

[40] Schenone AV, Culzoni MJ, Galera MM, Goicoechea HC (2013). Talanta.

[41] Gu J, Li H, Pei K, Cai H, Qin K, Zhang X, Zheng L, Liu X, Cai Y, Cai B (2014). J. Chromatogr. B.

[42] He MY, Deng YX, Shi QZ, Zhang XJ, Lv Y (2014). J. Ethnopharmacol..

[43] Yuan J, Wang Y, An R, Wang S, Li SJ, Jia JY, Bligh SWA, Wang XH, Ma YM (2012). J. Chromatogr. B.

[44] Zhan S, Shao Q, Fan X, Li Z (2015). Biomed. Chromatogr..

[45] Arancibia JA, Damiani PC, Escandar GM, Ibanez GA, Olivieri AC (2012). J. Chromatogr. B.

[46] Escandar GM, Goicoechea HC, Munoz de la Pena A, Olivieri AC (2014). Anal. Chim. Acta.

[47] Wu HL, Shibukawa M, Oguma K (1998). J. Chemom..

[48] Kamstrup-Nielsen MH, Johnsen LG, Bro R (2013). J. Chemom..

[49] Wang ZG, Wang SS, Sun YJ, Wang HY, Chen G, Wang XJ, Hattori M, Zhang HL (2014). J. Sep. Sci..

